# Pro-Ana and Pro-Mia keywords, their google search trend

**DOI:** 10.1192/j.eurpsy.2021.1860

**Published:** 2021-08-13

**Authors:** G. Lladó Jordan, M.D.C. Díaz García, P. Mediavilla Sánchez, B. Lozano Díez, J.A. Gómez Del Barrio, R. Ayesa-Arriola

**Affiliations:** Idival, Valdecilla Biomedical Research Institute, Santander, Spain

**Keywords:** eating disorders, Pro-Ana, Pro-Mia, google search trend

## Abstract

**Introduction:**

Eating Disorders (ED) have increased both in number of cases and diagnoses in recent years, partly due to the ease of searching on the Internet. This “community” as they call themselves has a proper language, which makes them easier to connect.

**Objectives:**

To know the search frequency of Pro-Ana and Pro-Mia terms in Spanish in the Google search engine.

**Methods:**

A manual screening was carried out based on the word analysis of Pro-Ana and Pro-Mia blogs to obtain their search frequency in Spanish. Using the Google Trends tool, a total of 19 word combinations related to ED and their advocacy were reviewed in the time period from 01/01/2019 to 01/12/20. Some of them such as: “carrera de kilos” (kilos race), “princesa de cristal” (glass princess), “princesa de porcelana” (porcelain princess) and “dieta ABC” (ABC diet) among other terms.

**Results:**

From 2019 to 2020 there has been an increase in the searches related to Eating Disorders (41.63%), ABC diet (9.72%), porcelain princess (25.52%) and kilos race (38.53%). There has also been a decrease in the search for thinspo ana (30.9%), tips ana (4.15%), blog mia (13.09%) or blog ana (0.79%).

**Conclusions:**

Search trends change over time as they meet the evolving needs. In several media we can find a clear increase in ED during this 2020 due to the confinement related to COVID-19. This is something that we can also relate to this increase in searches for some terms.

**Disclosure:**

No significant relationships.

